# High expression of *BCL3* in human myeloma cells is associated with increased proliferation and inferior prognosis

**DOI:** 10.1111/j.1600-0609.2009.01225.x

**Published:** 2009-05

**Authors:** Anne-Tove Brenne, Unn-Merete Fagerli, John D Shaughnessy, Thea Kristin Våtsveen, Torstein Baade Rø, Hanne Hella, Fenghuang Zhan, Bart Barlogie, Anders Sundan, Magne Børset, Anders Waage

**Affiliations:** 1 Department of Cancer Research and Molecular Medicine, Norwegian University of Science and TechnologyTrondheim, Norway; 2 Myeloma Institute for Research and Therapy, University of Arkansas for Medical SciencesLittle Rock, AR, USA; 3 Departments of Oncology, St. Olavs University HospitalTrondheim, Norway; 4 Departments of Immunology and Transfusion Medicine, St. Olavs University HospitalTrondheim, Norway; 5 Departments of Hematology, St. Olavs University HospitalTrondheim, Norway

**Keywords:** multiple myeloma, Bcl-3, nuclear factor-κB

## Abstract

**Background:**

*BCL3* is a putative oncogene encoding for a protein belonging to the inhibitory κB-family. We experienced that this putative oncogene was a common target gene for growth-promoting cytokines in myeloma cell lines.

**Methods:**

Gene expression of *BCL3* was studied in 351 newly diagnosed myeloma patients, 12 patients with smouldering myeloma, 44 patients with monoclonal gammopathy of undetermined significance and 22 healthy individuals. Smaller material of samples was included for mRNA detection by RT-PCR, protein detection by Western blot and immunohistochemistry, and for cytogenetic studies. A total of eight different myeloma cell lines were studied.

**Results:**

Bcl-3 was induced in myeloma cell lines by interleukin (IL)-6, IL-21, IL-15, tumor necrosis factor-α and IGF-1, and its upregulation was associated with increased proliferation of the cells. In a population of 351 patients, expression levels of *BCL3* above 75th percentile were associated with shorter 5-yr survival. When this patient population was divided into subgroups based on molecular classification, *BCL3* was significantly increased in a poor risk subgroup characterized by overexpression of cell cycle and proliferation related genes. Intracellular localization of Bcl-3 was dependent on type of stimulus given to the cell.

**Conclusion:**

*BCL3* is a common target gene for several growth-promoting cytokines in myeloma cells and high expression of *BCL3* at the time of diagnosis is associated with poor prognosis of patients with multiple myeloma (MM). These data may indicate a potential oncogenic role for Bcl-3 in MM.

Myeloma cells are dependent on signals from the bone marrow micro-environment for proliferation and survival (1). Myeloma cell growth factors mediate redundant effects since one growth factor can substitute for another *in vitro* (2, 3). This observation led us to hypothesize that intracellular signals generated by myeloma growth factors target common genes important for growth and survival of myeloma cells. These genes may represent potential drug targets. Microarray experiments done in our laboratory showed that the myeloma cell growth factors interleukin (IL)-6, IL-21 and tumor necrosis factor (TNF)-α all induced expression of the putative oncogene *BCL3* in the myeloma cell lines IH-1 and OH-2 (paper in preparation). These data supported previous studies done by Brocke-Heidrich *et al.* and Tsuyama *et al.*, who showed that *BCL3* is upregulated by IL-6 at mRNA level as well as at protein level in myeloma cell lines (4–6). However, the expression of *BCL3* in myeloma patient samples has not been studied. Based on this we decided to study the role of *BCL3* in primary myeloma cells as well as in myeloma cell lines in more detail.

*BCL3* was first identified through its involvement in the t (14;19) (q32;q13) translocation in B-cell chronic lymphocytic leukemia (CLL) (7). Leukemic cells from these patients had increased levels of *BCL3* mRNA, leading to the hypothesis that *BCL3* is a proto-oncogene contributing to leukemogenesis (8). *BCL3* is overexpressed in breast cancers, subtypes of lymphomas and nasopharyngeal carcinomas (9–12). Supporting the notion that *BCL3* is an oncogene, Viatour *et al.* demonstrated that overexpression of *BCL3* is sufficient to transform the mouse fibroblast cell line NIH3T3 and induce tumor growth subcutaneously in nude mice (13). Physiologically, *BCL3* has been implicated to play a role during B-cell development and as a negative regulator of immune responses (14–16).

*BCL3* encodes a protein denoted Bcl-3 that is a member of the inhibitory κB (IκB)-family (17, 18). The IκB proteins modulate the DNA-binding activity of nuclear factor-κB (NFκB), a family of transcription factors involved in apoptosis and cell growth (19). Activation of NFκB is implicated as an important mechanism for the development of antiapoptosis and drug resistance in multiple myeloma (20). Depending on context, Bcl-3 either activates or inhibits NFκB-dependent gene transcription through interactions with homodimers of NFκB p50 or p52 (14, 21–26). We here present evidence that *BCL3* is overexpressed in myeloma cells from a subset of myeloma patients, and that the high expression of *BCL3* at the time of diagnosis is associated with inferior prognosis. Furthermore, high expression of Bcl-3 in myeloma cell lines induced by growth-promoting cytokines is associated with increased proliferation of the cells. We propose that Bcl-3 contributes to regulation of NFκB-dependent gene transcription in myeloma cells, with potential oncogenic consequences.

## Materials and methods

### Cell lines and culture conditions

The human myeloma cell line ANBL-6 (gift from Dr D. Jelinek, Mayo Clinic, Rochester, MN, USA), IH-1 (3), INA-6 (gift from Dr M. Gramatzki, Erlangen, Germany), JJN-3 (gift from Dr J. Ball, Department of Immunology, University of Birmingham, UK), OH-2 (2), RPMI-8226 and U-266 (both from American Type Culture Collection, Rockville, MD, USA) were cultured as previously described (27). The CAG cell line (gift from Dr J Epstein, Little Rock, AK, USA) was grown in RPMI 1640 (Gibco, Paisley, UK) supplemented with l-glutamine 100 μg/mL, gentamicin 20 μg/mL (referred to as RPMI) and 10% heat inactivated fetal calf serum (FCS; HyClone, Logan, UT, USA). The cells were kept at 37°C in a humidified atmosphere containing 5% CO_2_, and were washed four times in Hanks` balanced salt solution (HBSS; Gibco, Paisley, UK) to deplete them of serum and cytokines before performing experiments.

### Patients and healthy individuals; separation of CD138^+^ plasma cells

Gene expression profiles (GEP) were studied in plasma cells from 351 patients with newly diagnosed multiple myeloma (MM), 12 patients with smouldering multiple myeloma (SMM), 44 patients with monoclonal gammopathy of undetermined significance (MGUS) and 22 healthy individuals. The patients were recruited from University of Arkansas for Medical Sciences, Little Rock, USA, and the use of patient samples in research studies was approved by the University of Arkansas for Medical Sciences (Little Rock, AR) Institutional Review Board. Details about classification of these patients have been previously described (28, 29).

We used patient samples from the Norwegian Research Biobank for multiple myeloma for studies of Bcl-3 expression by RT-PCR (10 patients) immunohistochemistry (18 patients with MM and three patients with plasmocytoma), Western blot (eight patients) and cIg-FISH (19 patients). The Norwegian Research Biobank for multiple myleoma has been approved by the Regional Ethics Committee of Middle Norway and Norwegian Health Authorities. All patients have signed informed consent to store samples in the Biobank and to use samples for research purposes. Separation of plasma cells before GEP was done as previously described (28).

### Cytokines and antibodies

IL-6 was purchased from Biosource (Camarillo, CA, USA). IL-10, IL-15 and insulin-like growth factor (IGF)-1 were from R&D systems (Abingdon, UK). TNF-α was from Genetech (South San Francisco, CA, USA), and IL-21 was a gift from R.Holly, ZymoGenetics (Seattle, WA, USA). Hepatocyte growth factor (HGF) was purified from a medium conditioned by JJN-3 as described previously (30). All cytokines except HGF were of recombinant human type. Polyclonal anti-Bcl-3 (sc-185), polyclonal anti-p65 (sc-109) and monoclonal anti-p50 (sc-8414) for Western blotting were purchased from Santa Cruz Biotechnologies (Santa Cruz, CA, USA). Monoclonal anti-GAPDH and anti-Lamin B used as loading controls on Western blots were purchased from Abcam (ab8245; Cambridge, UK) and Calbiochem (Cat#NA12; Darmstadt, Germany), respectively.

### Preparation of cRNA and microarray hybridization

RNA, cRNA preparation and hybridization to U133 Plus2 GeneChip microarrays (Affymetrix, Santa Clara, CA, USA) were performed as previously described (31).

### Statistical analysis

Gene expression was analyzed as previously described (28, 31). Differences in expression of *BCL3* between plasma cells from MM patients and normal plasma cells (NPC) were analyzed using One-Way anova test. Survival distributions were presented with the use of the Kaplan–Meier method and compared with the log-rank test. Statistical tests were performed with the software package spss 12.0 (SPSS, Chicago, IL, USA).

### Analysis of *BCL3* expression by quantitative real-time PCR (qPCR)

Plasma cells from 10 patients were isolated with CD138 antibodies conjugated to magnetic beads using a RoboSep (StemCell Technologies, Vancouver, BC, Canada) cell separation device. Total RNA isolated using the MirVana™ miRNA Isolation Kit (Applied Biosystems, Foster City, CA, USA). cDNA was synthesized using Taqman reverse transcriptase reagents from Roche-Applied Biosystems. qPCR of *BCL3* was performed using StepOne Real-Time PCR System, (Applied Biosystems). *BCL3* TaqMan primer (Hs00180403_m1, Taqman, Gene Expression Assays, Applied Biosystems) was used to detect *BCL3* expression. The comparative Ct-method was used for quantization with GAPDH (HS99999905_m1) as housekeeping gene.

### Western blotting

Cells (1.2 × 10^6^) were seeded in 3 mL RPMI supplemented with 0.1% bovine serum albumin (BSA, Sigma-Aldrich, St. Louis, MO, USA) before Western blotting of whole cell lysates. The ANBL-6, IH-1, INA-6 and OH-2 cell lines were starved overnight before they were stimulated with cytokines as indicated at time point 0 (T0). At this time point, cells that were not stimulated with cytokines were harvested. The viability of these cells as measured by propidium iodide-staining was 60–80%, similar to the viability of cells grown in IL-6 enriched medium. CAG, JJN-3, RPMI-8226 and U-266 were stimulated with IL-6 immediately after seeding or left without cytokines in RPMI with 0.1% BSA for 24 h. After harvesting, dry pellets were kept at −80°C until further processing. Pellets were lysed in lysis buffer (10% sodium dodecyl sulfate, 10 mm Tris-HCL, pH 6.8) at 50°C using a Hamilton injector, mixed with NuPage LDS sample buffer (Invitrogen, Carlsbad, CA, USA) containing 0.1 m dithiothreitol, and heated for 2 min at 90°C. The lysates (40 μg of proteins) were then separated on 10% NuPage Bis-Tris gels (Invitrogen), followed by electrophoretic transfer to nitrocellulose membranes (Bio-Rad, Hercules, CA, USA). Membranes were blocked in 50 mm Tris buffered saline pH 7.5 containing 0.05% Tween 20 and 5% non-fat dried milk (Nestle, Vevey, Switzerland) and incubated with antibodies as indicated. After incubation with horseradish peroxidase-conjugated secondary antibodies (DakoCytomation, Glostrup, Denmark), the proteins were visualized by ECL Western blotting detection reagents (Amersham Biosciences, Buckinghamshire, UK), scanned (Canoscan D1250 U25, Canon Europa NV, Amstelveen, The Netherlands) and imported into adobe photoshop elements 4.0 software (Adobe Systems Inc, San Jose, CA, USA) for image acquisition. Cytosol-and nuclear extracts of IH-1 were prepared using Active Motifs nuclear extract kit (Active Motif, CA, USA) according to the manufacturer’s protocol. These cells were seeded in RPMI containing 10% FCS and cytokines as indicated for 24 h without previous starvation of cells.

### Thymidine incorporation assays

To measure DNA synthesis, cells were seeded in triplets in 96-well cell culture plates (Corning Inc, Corning, NY, USA) at a density of 2 × 10^4^ cells per well in RPMI supplemented with 0.1% BSA and cytokines as indicated. ANBL-6, IH-1,INA-6 and OH-2 were pulsed with 0.75 μCi methyl-[^3^H]-thymidine (NEN Life Science products, Boston, MA, USA) after 30 h of stimulation with cytokines and harvested 18 h later using a Micromate 96-well harvester (Packard, Meriden, CT, USA). CAG, JJN-3, RPMI-8226 and U-266 were pulsed with 0.75 μCi methyl-[^3^H]-thymidine for 4 h after 24 h of stimulation. Beta-radiation was measured with Matrix 96 counter (Packard).

### Immunohistochemistry

Sections (4 μm) of formalin-fixed bone marrow biopsies were prepared and visualized as previously described (32). The sections were incubated with a monoclonal antibody specific for Bcl-3 (clone 1E8, Novocastra, Newcastle upon Tyne, UK) at a dilution of 1 : 25. Parallel sections were stained with anti CD138 (Dako, code no. M7228, clone MI 15, dilution 1 : 100). Only CD138^+^ cells were considered and the biopsies were defined as Bcl3^+^ when ≥20% of the CD138^+^ cells was stained.

### Cytoplasmic immunoglobulin fluorescence *in situ* hybridization (cIg-FISH)

Cytospin of Lymphoprep-separated (Dynal, Oslo, Norway) bone marrow mononuclear cells from MM patients were fixed in acetic acid/methanol (1:3 v/v, −20°C, 40 min) and air-dried. 5 μL of *BCL3* split probe (No.Y5411; DakoCytomation) was applied and the slide was sealed with coverslip and rubber cement. Hybridization was done in a Dako Hybridizer according to the manufacturer’s protocol. The cells were then incubated with 15% goat serum in phosphate buffered saline. Plasma cells were labeled with AMCA (7-amino-4-methylcoumarin-3-acetic acid)-conjugated anti-human IgG against cytoplasmic κ/λ-chains (Vector Laboratories, Burlingame, CA, USA). The slides were air dried in the dark before anti-fade (Vectashield had-set mounting medium without DAPI, Vector Laboratories) was added. The cells were scored as previously described (32). Normal locus was detected as a colocalization of red and green signal, and a split was detected as separate red and green signal.

### Assessment of NFκB activity

Activation of the NFκB family members can be measured by their capacity to bind to consensus DNA binding sites (33). We used the TransAm NFκB p65 and p50 transcription Factor Assay Kit (Active Motif, CA, USA) to quantify the DNA binding activity of p50 and p65. The assay was performed according to the manufacturer’s protocol. Briefly, IH-1 cells were cultured for 24 h without previous starvation in RPMI containing 10% FCS and cytokines as indicated. Nuclear extracts were prepared and incubated in 96-well plates coated with an immobilized oligonucleotide containing the 5`-GGACTTTCC-3` consensus binding site for NFκB. Hybridization to the target oligonucleotide was detected by incubation with primary antibodies specific for p65 of p50, visualized by anti-IgG1-HRP and quantified at 450 nm. Specificity was confirmed by incubating with a wild type consensus oligonucleotide that competed with the substrate for binding to the immobilized oligonucleotide.

## Results

### Growth promoting cytokines induce Bcl-3 in myeloma cell lines

**Figure 1:**
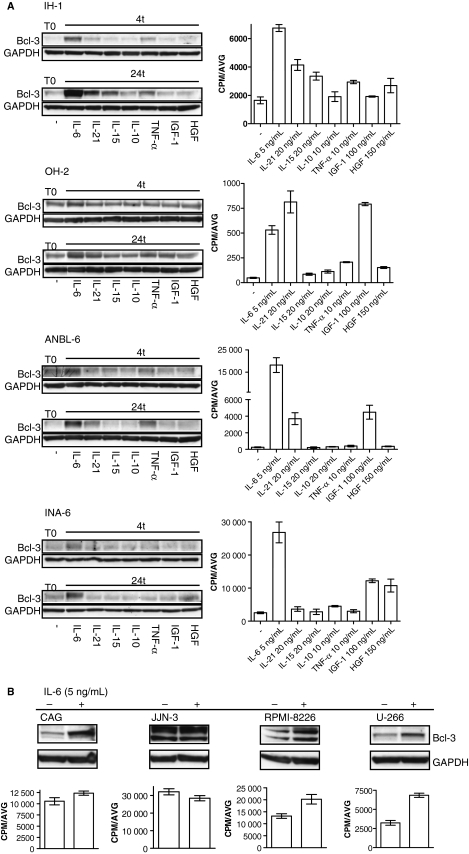
Protein expression of Bcl-3 in myeloma cell lines and corresponding cell proliferation after cytokine stimulation. Bcl-3 expression in whole cell lysates and corresponding myeloma cell proliferation. The same cytokine concentrations were used for Western blot as for [^3^H]-thymidine incorporation assay. Average counts per minute (CPM/AVG) are plotted along the *y*-axis on graph. Bars represent mean + SD of triplicate wells. (A) IL-6 dependent myeloma cell lines (IH-1, OH-2, ANBL-6, INA-6) were harvested after overnight starvation (T0) or stimulated with cytokines as indicated for 4 and 24 h. (B) IL-6-independent myeloma cell lines (CAG, JJN-3, RPMI-8226, U-266) were stimulated with IL-6 or left unstimulated for 24 h. GAPDH was used as loading control.

We stimulated the IL-6-dependent myeloma cell lines ANBL-6, IH-1, INA-6 and OH-2 with cytokines known to promote growth of myeloma cells (2, 3, 34–37). The cytokines were given in optimal concentrations for growth. Protein expression of Bcl-3 increased in these cell lines after 4 h of cytokine stimulation compared with baseline level, and increased even more after 24 h of stimulation (Fig. 1A). IL-6, IL-21 and TNF-α were the most potent inducers of Bcl-3. Cytokines that induced Bcl-3 also induced proliferation of the corresponding myeloma cell line (Fig. 1A). However, there were a few exceptions from this association. In ANBL-6, TNF-α increased the level of Bcl-3 without stimulating proliferation, whereas IGF-1 induced proliferation without increasing Bcl-3 expression. We also tested the effect of IL-6 on Bcl-3 expression in the IL-6 independent cell lines CAG, RPMI-8226, U-266 and JJN-3. Bcl-3 was induced in the cell lines where IL-6 gave a proliferative response. JJN-3 had constitutive expression of Bcl-3, and in this cell line IL-6 had no effect on Bcl-3 expression (Fig. 1B).

### Expression of *BCL3* in CD 138^+^ bone marrow plasma cells from MM patients is associated with poor outcome

**Figure 2:**
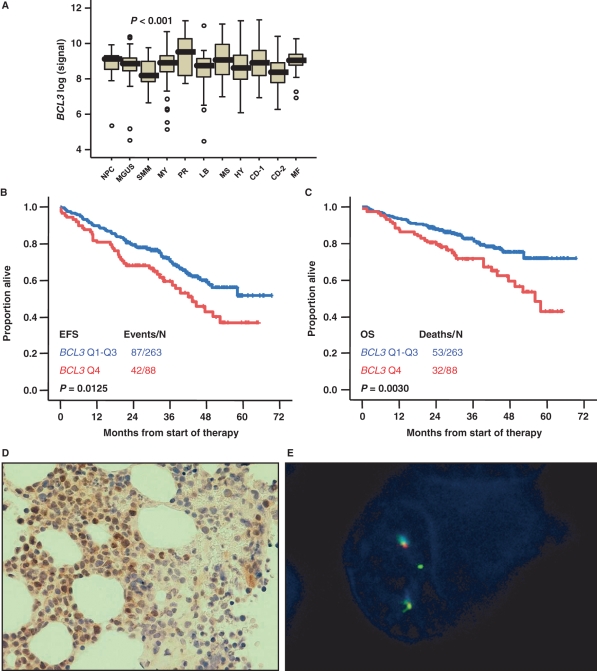
Expression of *BCL3* and its protein in CD138^+^ MM plasma cells. (A) Box plot of *BCL3* expression in NPC and in plasma cells from patients with MGUS, SMM and overt MM. The expression level of *BCL3* is in addition shown for subgroups of MM patients based on molecular classification. Sample groups are along the *x*-axis and the natural log transformed Affymetrix-derived signal is plotted on the *y*-axis. The top, bottom and middle lines of each box correspond to the 75th percentile (top quartile), 25th percentile (bottom quartile) and 50th percentile (median), respectively. The whiskers extend from the 10th percentile (bottom decile) and 90th percentile (top decile). Open circles denote outliers within each group. The *P*-value states that *BCL3* was significantly increased in the proliferation subgroup (PR) compared with the other subgroups as well as to the NPC, MGUS and SMM groups. MY, all MM patients; LB, low bone disease subgroup; MS, MMSET subgroup; HY, hyperdiploid subgroup; CD-1, CCND1 subgroup: CD-2, CCND3 subgroup: MF, MAF/MAFB subgroup. (B) and (C) Kaplan–Meier 5 yr estimates of EFS and OS showed inferior EFS and OS of MM patients with *BCL3* expression levels above 75th percentile(top quartile). (D) Bcl-3 was detected in bone marrow biopsies by immunohistochemistry. The bone marrow biopsy illustrated here had an overall 20% Bcl-3^+^ plasma cells. (E) MM patient with an unbalanced *BCL3* translocation detected by cIg-FISH. The red signal is upstream and the green signal is downstream of the *BCL3* gene. Colocalization of the signals represents normal genes, while a single green signal represents an unbalanced translocation.

We studied GEP of CD138^+^ bone marrow plasma cells from 351 newly diagnosed MM patients, 22 healthy individuals, 44 patients with MGUS and 12 patients with SMM. Based on ‘present’ detection call, *BCL3* was present in 229 (65%) of MM plasma cell samples and in 11 (46%) of normal plasma cell samples (NPC; data not shown). By further comparison of *BCL3* expression between NPC and primary MMs based on absolute intensity signal, there was no significant difference in *BCL3* expression (median 445 (range: 23–2555) in MM plasma cells vs. median 534 (range: 41–970) in NPC, *P* = 0.7). However, when the expression of *BCL3* among NPC, MGUS, SMM, and MM subgroups was compared, the level of *BCL3* was significantly increased in one of the gene expression-defined high-risk subgroups, namely the PR (proliferation) subgroup (Fig. 2A) (28). When looking at outcome, Kaplan–Meier analysis revealed 5-yr event-free survival estimates of 37% (EFS, *P* = 0.0125) and 5-yr overall survival estimates of 42% (OS, *P* = 0.0030) among 88 patients with *BCL3* expression above 75th percentile, compared with 52% and 72% among the other 263 patients respectively (Figs 2B and 2C). Our study population of myeloma patients was uniformly treated with tandem transplants (28).

### Bcl-3 is detected at mRNA and protein level in plasma cells from MM patients

Expression of *BCL3* mRNA was studied by qPCR in samples from 10 randomly selected MM patients (Figure S1). All patients expressed *BCL3*, with six of the patients expressing four times or more the amount compared to the patient with the lowest expression level, confirming the Affymetrix data showing that a subset of MM patients expresses high amounts of *BCL3*. Expression of Bcl-3 at protein level was examined by two independent methods, immunohistochemistry in bone marrow biopsies and Western blot of purified myeloma cells. Of 18 newly diagnosed MM patients and in biopsies from three plasmocytoma patients, two biopsies (one bone marrow biopsy from a MM patient and one plasmocytoma biopsy) were positive for Bcl-3, with 20% and 40% Bcl-3+plasma cells, respectively (Fig. 2D). In addition, one MM bone marrow biopsy had 5–10% of Bcl3^+^ plasma cells. When looking at the Bcl-3 protein expression in CD138^+^ plasma cells from eight myeloma patients, we detected Bcl-3 in seven of eight samples with Western blot (data not shown).

### The *BCL3* gene is altered in malignant plasma cells from MM patients

To reveal whether alterations in the *BCL3* gene locus could explain the upregulation of Bcl-3 in malignant plasma cells from MM patients, we examined bone marrow aspirates from 19 newly diagnosed MM patients using cIg-FISH and a commercial *BCL3* split probe. We found that four patients had an extra copy of the *BCL3* gene (data not shown). In addition, one t (4;14)^+^ patient had an unbalanced translocation involving an extra copy of the *BCL3* locus (Fig. 2E).

### Intracellular localization of Bcl-3 is dependent on type of stimulus given to the cell

**Figure 3:**
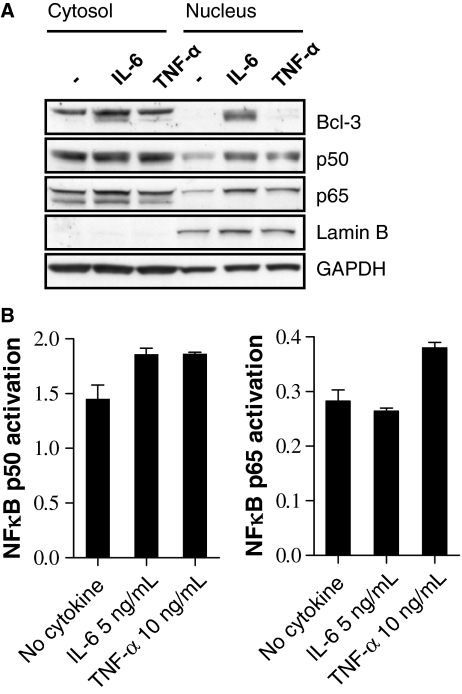
Intracellular localization of Bcl-3 and activation of NFκB p50 and p65**.** (A) Bcl-3 was detected on Western blot in the nuclear fraction of IL-6-stimulated IH-1 cells, but was absent in the nuclear fraction of TNF-α- and unstimulated cells. The NFκB proteins p50 and p65 were present in all conditions. GAPDH was used as loading control. Presence of GAPDH in the nucleus is earlier reported (43). Lamin B was used to determine the purity of the extracts. (B) Activation of NFκB p50 in nuclear extracts of IH-1 after IL-6 stimulation, and p50 and p65 after TNF-α-stimulation. Bars represent mean + SD of duplicate wells. The *y*-axis denotes optical density (OD) at 450 nm. The experiments were repeated three times.

To be activated, Bcl-3 is translocated to the nucleus of the cells (26). We wanted to study if Bcl-3 is a nuclear protein in myeloma cells. We used IH-1 as a model cell line since Bcl-3 was highly inducible by cytokines in this cell line (Fig. 1A). After stimulation with IL-6, Bcl-3 was detected in the nuclear fraction of IH-1 (Fig. 3A). By contrast, Bcl-3 was detected only in the cytosolic fraction of TNF-α-stimulated cells. Both IL-6 and TNF-α have a proliferative effect on myeloma cells (Fig. 1A).

Bcl-3 has been shown to associate with homodimers of NFκB p50 or p52 in the nucleus and repress DNA-binding of p50/p65, the classical NFκB dimer activated by TNF-α (14, 21–26, 38). We therefore examined the intracellular localization of NFκB p50 and p65 in IH-1 after IL-6 and TNF-α stimulation, as well as their DNA-binding capacity under these conditions. We found that both these proteins were present in the nucleus after TNF-α and IL-6 stimulation (Fig. 3A). However, while TNF-α activated both p65 and p50, IL-6 activated p50 only (Fig. 3B). Hence, after IL-6 stimulation, p50 was activated, and this activation coincided with nuclear presence of Bcl-3. Simultaneously, there was a lack of p65 activation.

## Discussion

This work shows for the first time that myeloma cells from MM patients express *BCL3* and that high expression of *BCL3* at the time of diagnosis is associated with reduced survival. Furthermore, in a comparison of molecularly defined subgroups, *BCL3* expression was significantly increased in a high risk subgroup characterized by overexpression of cell cycle-and proliferation-related genes (28). Our data are consistent with the concept that high *BCL3* expression is an event associated with poor outcome.

We have not shown that *BCL3* is an independent adverse prognostic factor and the exact relevance of elevated expression of *BCL3* in myeloma high-risk disease is currently unclear. Interestingly, Annunziata *et al.* has shown that the high risk subgroup with high expression of *BCL3* has a very low average expression of NFκB signature genes (39). NFκB signature genes are genes that are activated by the classical and/or alternative NFκB pathway (39). It is therefore tempting to speculate that the nuclear presence of Bcl-3 as after IL-6 stimulation activates a different set of genes than activation of the classical and/or alternative NFκB pathway in myeloma cells.

The array data was confirmed with qPCR in a material with 10 patients. In another randomly selected material with eight patients with MM, Bcl-3 protein was detected in seven of eight samples by Western blot. We also examined the expression of Bcl-3 at protein level by immunohistochemical staining of biopsies from MM patients and patients with plasmacytoma, and found that 11% (two of 18) of the biopsies stained positive for Bcl-3. This proportion of Bcl-3^+^ plasma cell malignancies is similar to the proportion of B-cell lymphomas expressing Bcl-3 (6%) (9). The discrepancy between the proportion of patient samples positive for *BCL3* at mRNA level and biopsies positive for Bcl-3 at protein level may be caused by lower sensitivity of immunostaining of biopsies, a well-known problem when using immunohistochemistry as detection method. However, the number of patient samples tested was small, and we cannot rule out that there is reduced translation or post-translational modifications.

Our data showed that stimulation of myeloma cells with various cytokines well-known to promote proliferation increased Bcl-3 expression. These data indicate that the gene encoding Bcl-3 is indeed a common target gene for growth promoting cytokines in myeloma cells. As growth-promoting cytokines are frequently present in the bone marrow of MM patients, cytokine signaling is a highly possible mechanism for upregulation of Bcl-3 in myeloma cells *in vivo*. Importantly, and in concordance with the patient data, we found that the induction of Bcl-3 by cytokines was associated with increased proliferation of myeloma cells. However, in line with the results from Brocke-Heidrich *et al.* (5), we could not show any effect on thymidine incorporation or apoptosis when down regulating Bcl-3 with siRNA in the INA-6 cell line (data not shown). These results are from only one cell line and further studies are needed to clarify the exact role of Bcl-3.

Additional potential mechanisms for upregulation of Bcl-3 in MM patients are gains/amplification of the *BCL3* gene, as described in patients with anaplastic large cell lymphoma, or translocations involving chromosome 19, as found in patients with CLL (40, 41). Our studies substantiate that both these mechanism may be active in freshly isolated MM patients cells. The *BCL3* gene is located at chromosome 19, a chromosome with frequent trisomy in MM patients having a hyperdiploid tumor (42).

In our study, intracellular localization of Bcl-3 was dependent of type of stimulus given to the cell. Nuclear accumulation of Bcl-3, as after IL-6 stimulation, has been shown to be associated with p50/Bcl-3- or p52/Bcl-3 dependent gene activation and subsequent proliferation in *CYLD*-lacking keratinocytes (26). It is possible that similar mechanisms are active in myeloma cells, and may explain why we observed activation of p50 in IH-1 after IL-6 stimulation.

The starting point of this study was our observation that IL-6, IL-21 and TNF-α induced expression of *BCL3* in the myeloma cell lines OH-2 and IH-1. Our study demonstrates that also the protein, Bcl-3, is produced in cell lines by cytokine stimulation and is associated with increased proliferation of the cells. We show for the first time that Bcl-3 is present in a subset of MM patients, and that the high gene expression at the time of diagnosis is associated with inferior prognosis as demonstrated in a large cohort of newly diagnosed patients. Our results indicate a potential role for Bcl-3 in the development of multiple myeloma, and further studies are needed to clarify this.

## Supporting Information

**Figure S1.** *BCL3* mRNA expression in samples from 10 MM patients.
