# Supplementary figures and images for: High expression of BCL3 in human myeloma cells is associated with increased proliferation and inferior prognosis
Source: Eur J Haematol. 2009 May;82(5):354–63. doi: 10.1111/j.1600-0609.2009.01225.x (PMC2704939; doi:10.1111/j.1600-0609.2009.01225.x)

## Slide 1
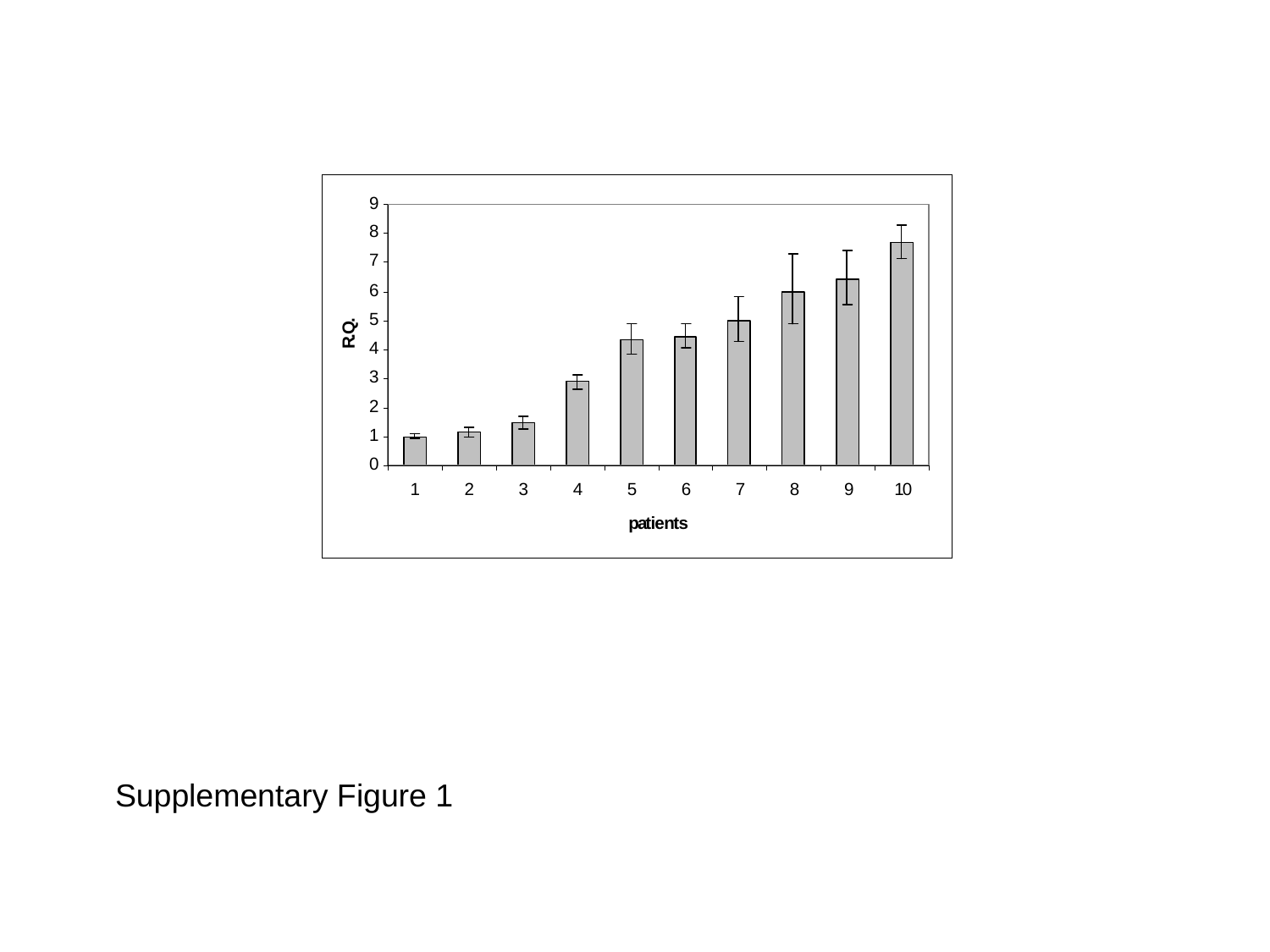

Supplementary Figure 1

Supplement: Supplementary file 1 [file ejh0082-0354-SD1.ppt]
